# Cracking of Rubber Plates

**Published:** 1867-01

**Authors:** Gam’l Jackson


### CRACKING OF RUBBER PLATES.

BY GAM'L JACKSON.

The liability of metallic plates, after being worn in the mouth for a considerable time, to crack is well known. Rubber Plates though more elastic are less tenacious, and are thus liable to the same accident. It is easy to understand how the combined force of mastication and the wedging of some hard substance between the teeth may start a plate ; a start once effected the destruction of the plate is sure to follow.

I have made over, within two years, three upper plates of this kind ; one of them made originally by myself. One of the others was a heavy, well vulcanized and perfectly finished piece of work, and was worn by a gentleman of unquestionable veracity, who told me that the plate had not met with any accident, but had broken simply by eating with it.

As a protection against such breakage I now imbed a double headed bolt in the plate, near to the pins of the centrals, I make the bolts from hard gold wire, nearly double the thickness of the pins in plate teeth, and long enough to reach beyond the first pin in the centrals. The best way to head the bolts is to punch a hole the size of the bolt near the end of a strip of plate, about half an inch long and as wide as the desired diameter of the head, and solder on to the wire. The bolt thus made should be pressed in the wax to the place it is intended to occupy in the rubber, just before the piece is put into the flask. The strips extending from the heads will be imbedded in the investing plaster, and will prevent any displacement of the bolt during the process of "packing."

If no other dentist has experienced the above mentioned accident, then the aspiring author of this article is abased ; but it may still be claimed that it was not written wholly for vainglorious ostentation, and that being in some degree original, it does not look like an expiring flicker of the literature of a science—apologies which cannot reasonably be offered for several additions to the dental literature, bound and otherwise.

